# Supplementary material for: Splice-Junction-Based Mapping of Alternative Isoforms in the Human Proteome
Source: Cell Rep. Author manuscript; Available in PMC 2020 Jan 15. (PMC6961840; doi:10.1016/j.celrep.2019.11.026)
Supplement: 2 [file NIHMS1546469-supplement-2.zip › DF1/PXD009737/TestisLysC/TestisLysC_15_ART3_LEDHAPGPVPVPGPK.pdf]

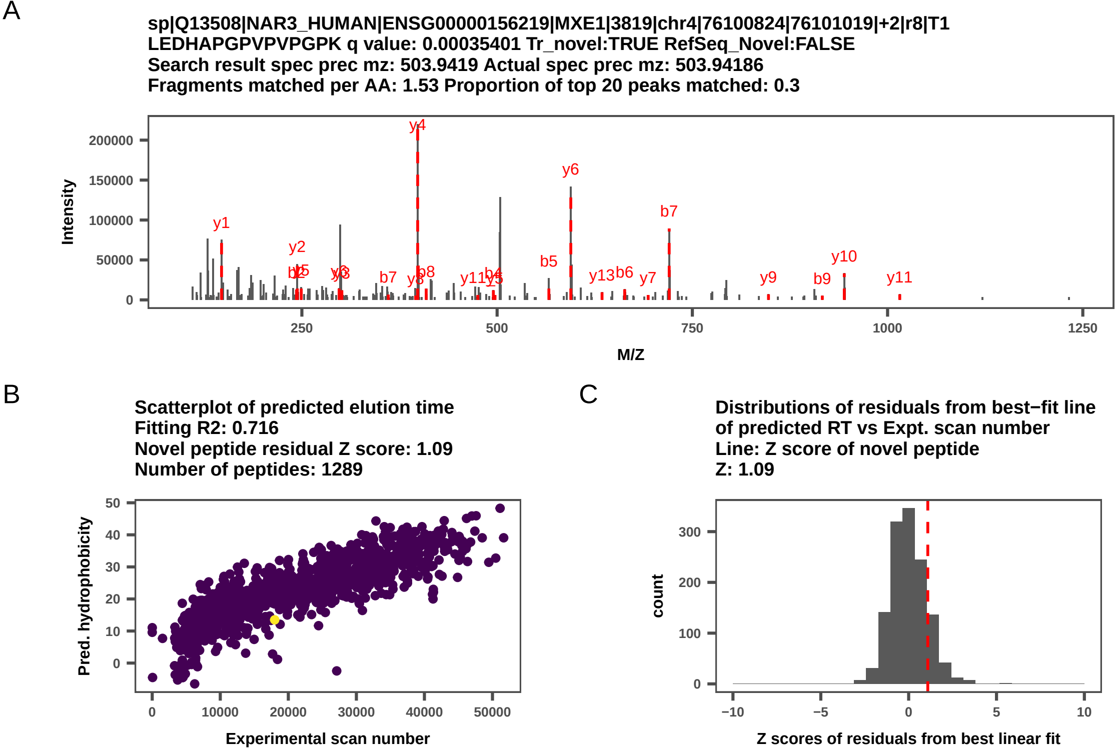

A
sp|Q13508|NAR3_HUMAN|ENSG00000156219|MXE1|3819|chr4|76100824|76101019|+2|r8|T1
LEDHAPGPVPVPGPK q value: 0.00035401 Tr_novel:TRUE RefSeq_Novel:FALSE
Search result spec prec mz: 503.9419 Actual spec prec mz: 503.94186
Fragments matched per AA: 1.53 Proportion of top 20 peaks matched: 0.3
Intensity
200000
150000
100000
50000
0
y1
y2
y4
b7
b8
b5
y6
b6
y7
b7
y9
b9
y10
y11
250
500
750
1000
1250
M/Z
B
Scatterplot of predicted elution time
Fitting R2: 0.716
Novel peptide residual Z score: 1.09
Number of peptides: 1289
Pred. hydrophobicity
50
40
30
20
10
0
10000
20000
30000
40000
50000
Experimental scan number
C
Distributions of residuals from best−fit line
of predicted RT vs Expt. scan number
Line: Z score of novel peptide
Z: 1.09
count
300
200
100
0
−10
−5
0
5
10
Z scores of residuals from best linear fit
